# Transanal total mesorectal excision (taTME) for rectal cancer: a systematic review and meta-analysis of oncological and perioperative outcomes compared with laparoscopic total mesorectal excision

**DOI:** 10.1186/s12885-016-2428-5

**Published:** 2016-07-04

**Authors:** Bin Ma, Peng Gao, Yongxi Song, Cong Zhang, Changwang Zhang, Longyi Wang, Hongpeng Liu, Zhenning Wang

**Affiliations:** Department of Surgical Oncology and General Surgery, the First Hospital of China Medical University, Shenyang, 110001 People’s Republic of China

**Keywords:** Transanl TME, Laparoscopic TME, Rectal cancer, Short-term outcomes

## Abstract

**Background:**

Transanal total mesorectal excision (taTME) is an emerging surgical technique for rectal cancer. However, the oncological and perioperative outcomes are controversial when compared with conventional laparoscopic total mesorectal excision (laTME).

**Methods:**

A systematic review and meta-analysis based on Preferred Reporting Items for Systematic Reviews and Meta-analyses (PRISMA) guidelines was conducted in PubMed, Embase and Cochrane database. All original studies published in English that compared taTME with laTME were included for critical appraisal and meta-analysis. Data synthesis and statistical analysis were carried out using RevMan 5.3 software.

**Results:**

A total of seven studies including 573 patients (taTME group = 270; laTME group = 303) were included in our meta-analysis. Concerning the oncological outcomes, no differences were observed in harvested lymph nodes, distal resection margin (DRM) and positive DRM between the two groups. However, the taTME group showed a higher rate of achievement of complete grading of mesorectal quality (OR = 1.75, 95% CI = 1.02–3.01, *P =* 0.04), a longer circumferential resection margin (CRM) and less involvement of positive CRM (CRM: WMD = 0.96, 95% CI = 0.60–1.31, *P <*0.01; positive CRM: OR = 0.39, 95% CI = 0.17–0.86, *P =* 0.02). Concerning the perioperative outcomes, the results for hospital stay, intraoperative complications and readmission were comparable between the two groups. However, the taTME group showed shorter operation times (WMD = –23.45, 95% CI = –37.43 to –9.46, *P <*0.01), a lower rate of conversion (OR = 0.29, 95% CI = 0.11–0.81, *P =* 0.02) and a higher rate of mobilization of the splenic flexure (OR = 2.34, 95% CI = 0.99–5.54, *P =* 0.05). Although the incidence of anastomotic leakage, ileus and urinary morbidity showed no difference between the groups, a significantly lower rate of overall postoperative complications (OR = 0.65, 95% CI = 0.45–0.95, *P =* 0.03) was observed in the taTME group.

**Conclusions:**

In comparison with laTME, taTME seems to achieve comparable technical success with acceptable oncologic and perioperative outcomes. However, multicenter randomized controlled trials are required to further evaluate the efficacy and safety of taTME.

## Background

Rectal cancer ranks as one of the most common types of carcinoma throughout the world [1]. Over recent decades, total mesorectal excision (TME) performed by an open approach has become the standard technique for the surgical treatment of rectal cancer [2]. Over time, to achieve a minimally invasive surgical treatment, TME has shifted from the open approach to a laparoscopic technique. Recently published randomized clinical trials (RCTs), such as COLOR II, COREAN and CLASICC, have shown better results for laparoscopic total mesorectal excision (laTME), in terms of short-term and long-term outcomes, when compared with open TME [3–6]. However, the utility of laTME is limited in patients with low rectal cancer, who require surgeons with experience in ultra-low sphincter-saving laparoscopic surgery, which has a high risk of leaving a positive circumferential resection margin (CRM). In addition, narrow pelvic anatomy, male sex and high body mass index (BMI) are also unfavorable patient characteristics for a laparoscopic approach [7]. Furthermore, because of the limited view of the distal margin of the tumor, conversion rates to open procedures remain unsatisfactory [8, 9]. The pressing need to overcome these challenges has motivated surgeons to develop alternative techniques for treatment of rectal cancer, especially for patients with mid- and low-rectal lesions.

Based on the aforementioned considerations, the concept of a “down-to-up” procedure and transanal TME (taTME) has been proposed to give a new option in cases where laTME is difficult. In fact, taTME is not a completely novel concept and it has benefited from previous experience of transabdominal–transanal (TATA) operations, transanal endoscopic microsurgery (TEM), transanal minimally invasive surgery (TAMIS) and natural orifice transluminal endoscopic surgery (NOTES) [10–12]. Since the first taTME resection assisted by laparoscopy was reported in 2010 [13], taTME performed on patients with rectal cancer has showed promising results with regard to pathological quality, and short- and mid-term outcomes [14–16]. Although taTME may improve the distal mesorectal dissection, which is the most technically challenging part of a transabdominal TME, whether the oncological and perioperative outcomes of taTME are better than those of laTME remains controversial. Hence, a quantitative analysis was necessary to provide direct evidence of the benefits of taTME.

Therefore, this meta-analysis was conducted to compare the oncological and perioperative outcomes of taTME and laTME for patients with mid- and low-rectal cancer.

## Methods

### Search strategy

This systematic review and meta-analysis were conducted in accordance with Preferred Reporting Items for Systematic Reviews and Meta-analyses (PRISMA) guidelines (http://www.prisma-statement.org/) [17]. A comprehensive search of published studies was performed in PubMed, Embase and the Cochrane Database (from January 2010 to November 2015). The MeSH and main keywords were as follows: “transanal”, “transanal total mesorectal excision” or “taTME”, “transanal minimally invasive surgery” or “TAMIS”, “transanal endoscopic microsurgery” or “TEM”, “natural orifice transluminal endoscopic surgery” or “NOTES”, “perineal approach”, “rectal cancer” and “proctectomy”. Based on these MesH and main keywords, we formulated the search strategy (for PubMed) as following: (transanal OR transanal minimally invasive surgery OR TAMIS OR transanal endoscopic microsurgery OR TEM OR transanal specimen extraction OR natural orifice specimen extraction OR NOSE OR natural orifice transluminal endoscopic surgery OR NOTES OR peritoneal) AND (total mesorectal excision OR TME OR proctectomy) AND rectal. All the relevant studies which described a comparison between taTME and laTME were checked carefully (including the reference lists of relevant studies). All studies were restricted to the English language.

### Inclusion and exclusion criteria

According to the PICOS criteria (population, intervention, comparison, outcomes and study design), studies were selected in our present meta-analysis according to the following eligibility criteria: (1) population: patients were definitely diagnosed with rectal cancer; (2) intervention: surgical treatment for rectal cancer (taTME/laTME); (3) comparison: taTME versus laTME; (4) outcomes: oncological and perioperative outcomes compared between two groups; (5) study design: randomized controlled trials, cohort trials or matched case–control trials with sample size greater than 20. The exclusion criteria were: (1) no laTME group as a control; (2) absence of the outcomes of interest; (3) duplicate publication or provision of insufficient data. All the studies included were checked carefully once again to avoid the inclusion of studies which were based on the same database or patient population as another included report.

### Data extraction and assessment of the risk of bias

Two reviewers (B Ma and P Gao) reviewed and assessed each of the included studies. In addition, data extraction was performed independently, and the following information was collected: (1) study characteristics: first author, year of publication, country, study type (RCT/cohort trial/matched case–control trial) and number of patients enrolled; (2) patient baseline: sex, age, tumor site (mid/low), tumor location (distance above the anal verge), body mass index, neoadjuvant treatment, American Society of Anesthesiologists (ASA), pT stage and pN stage; (3) study design: surgical type of taTME (partial/total), oncological outcomes (quality of mesorectum, harvested lymph nodes, CRM, positive CRM, distal resection margin (DRM), positive DRM and perioperative outcomes (operation time, conversion, mobilization of splenic flexure, hospital stay, intraoperative complications, postoperative complications and readmission). The Newcastle–Ottawa Scale (NOS) criterion was used to evaluate the quality of the studies included [18]. All disagreements were resolved by discussion between the two reviewers (B Ma and P Gao).

### Statistical analysis

In this meta-analysis, continuous variables representing the oncological and perioperative outcomes were analyzed by the weighted mean difference (WMD). If the study did not provide values for the mean and standard deviation (SD), we used the method of Hozo et al. to calculate the mean and SD for our overall analysis [19]. We used odds ratios (ORs) to evaluate the dichotomous variables for the oncological and perioperative outcomes. In addition, the Q test and I^2^ statistic were used to evaluate heterogeneity among studies. A Cochrane Q statistical *P* value <0.10 and/or I^2^ > 50% was taken to indicate significant heterogeneity, and in this case a random-effects model was used for the pooled analysis [20, 21]. Otherwise, a fixed-effects model was employed. All statistical values were computed with 95% confidence intervals (CI), and the two-tailed *P* value threshold for statistical significance was set at 0.05. Furthermore, based on the surgical type of taTME, we conducted a subgroup analysis to explore further the advantages of total taTME using a laparoscopic approach. Finally, publication bias was tested using funnel plots. All the statistical analyses were performed using software from the Cochrane Collaboration (RevMan v5.3; Nordic Cochrane Centre).

## Results

### Selected studies

**Fig. 1:**
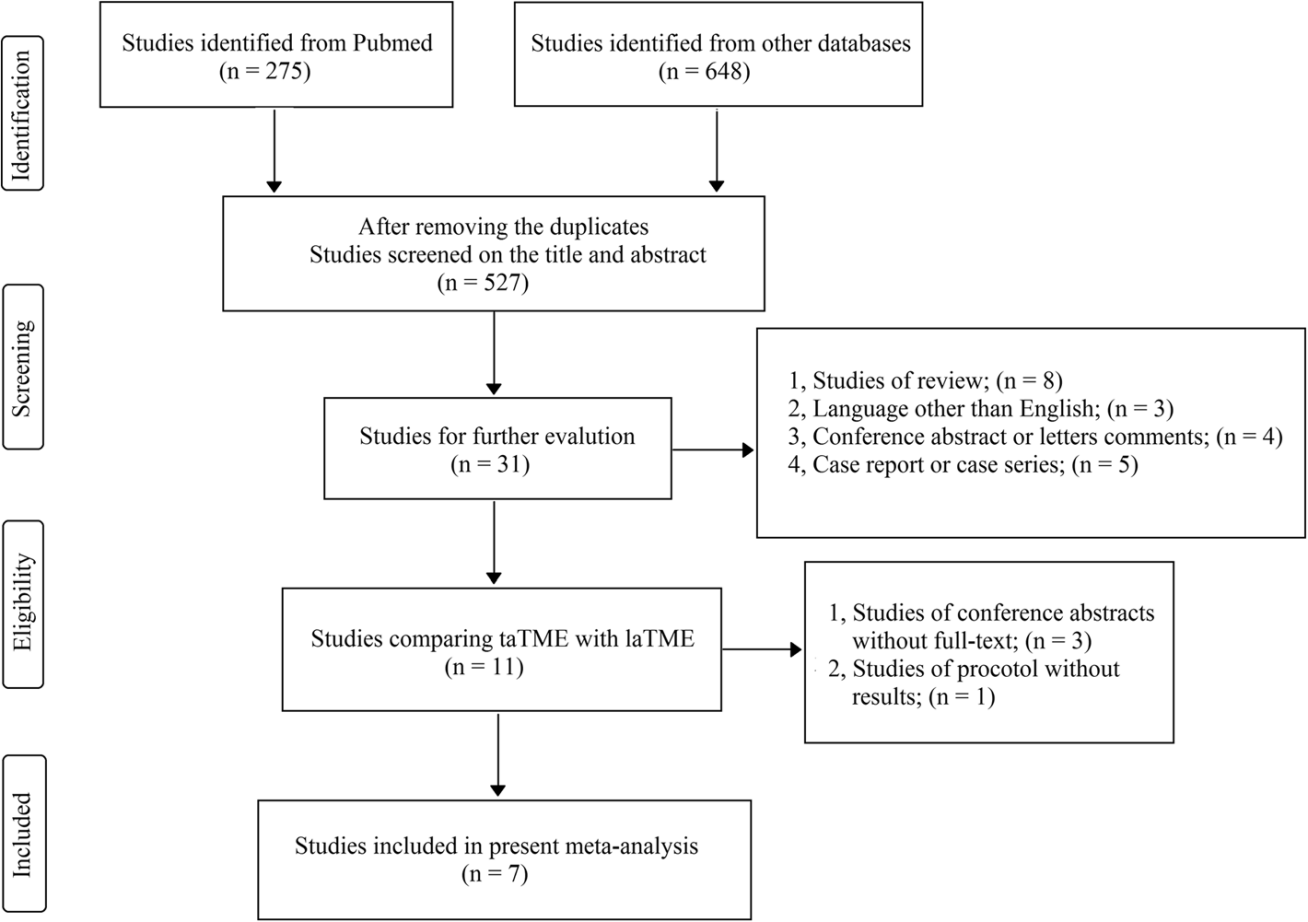
Flow chart showing the selection process for the included studies

The search strategy initially identified 923 studies (Pubmed = 275; other databases = 648). After exclusion of duplicates and irrelevant studies, 11 potentially relevant studies were obtained for further assessment. Among these studies, three studies were conference abstracts from which we could not extract sufficient information for our final analysis [22–24]. In addition, one report described a protocol for a multicenter RCT comparing transanal TME and laTME for mid- and low-rectal cancer [25]. Finally, seven studies including 573 patients were included our meta-analysis (taTME group = 270; laTME group = 303) [26–32]. A flow chart of the search strategies, which includes the reasons for exclusion of studies, is illustrated in Fig. 1. The seven studies were from France, the Netherlands, Taiwan, Spain and Denmark. The study characteristics, patient baseline data and methodological quality assessment scores of the studies included are summarized in Table 1.

**Table 1:** Baseline characteristics of the included studies

Studies (NOS score)	Year	Country	Study design	Gender Male/Female	BMI Mean ± SD/median (range)		Age Mean ± SD/median (range)		ASA I + II/III + IV		Tumor location	Neoadjuvant treatment (Yes/No)		taTME type
				taTME laTME	taTME	laTME	taTME	laTME	taTME	laTME		taTME	laTME	
Velthuis [31] (3) 2014		Netherland	MCC	18/7 18/7	25 (20-36)	28 (21-34)	NR	NR	NR	NR	low/mid	25/0	25/0	Total
Kanso [27] (6)	2015	France	MCC	36/15 26/8	24 ± 4	24 ± 4	59 ± 11	59 ± 11	47/4	31/3	low	43/41^a^	28/27^a^	Partial
Hevia [28] (4)	2014	Spain	MCC	24/13 22/15	23.7 ± 3.6	25.1 ± 4.0	64.5 ± 11.8	69.5 ± 10.5	30/7	25/12	low/mid	28/9	23/14	Total
Chen [30] (4)	2015	Taiwan	MCC	38/12 76/24	24.2 ± 3.7	24.6 ± 3.1	57.3 ± 11.9	58.3 ± 11.3	33/17	69/31	low/mid	50/0	100/0	Total
Denost [32] (6) 2014		France	RCT	37/13 32/18	25 (17-33)	26 (18-38)	64 (39-82)	63 (31-90)	49/1	49/1	low	40/10	44/6	Partial
Perdawood [26] (4) 2015		Denmark	MCC	19/6 19/6	28 (18-46)	26 (19-38)	70 (54-76)	70 (49-84)	19/6	22/3	low/mid	7/18	4/21	Total
Angelis [29] (4) 2015		France	MCC	21/11 21/11	25.2 ± 3.5	24.5 ± 3.2	64.9 ± 10.0	67.2 ± 9.6	31/1	31/1	low/mid	27/5	23/9	Total

*taTME* transanal total mesorectal excision, *laTME* laparoscopic total mesorectal excision, *BMI* body mass index, *ASA* American Society of Anesthesiologists, *MCC* matched case control, *RCT* randomized controlled trial,

^a^In taTME group, 43 patients received neoadjuvant radiotherapy and 41 patients received neoadjuvant chemotherapy. In laTME group, 28 patients received neoadjcpuvant radiotherapy and 27 patients received neoadjuvant chemotherapy

### Oncological outcomes

**Fig. 2:**
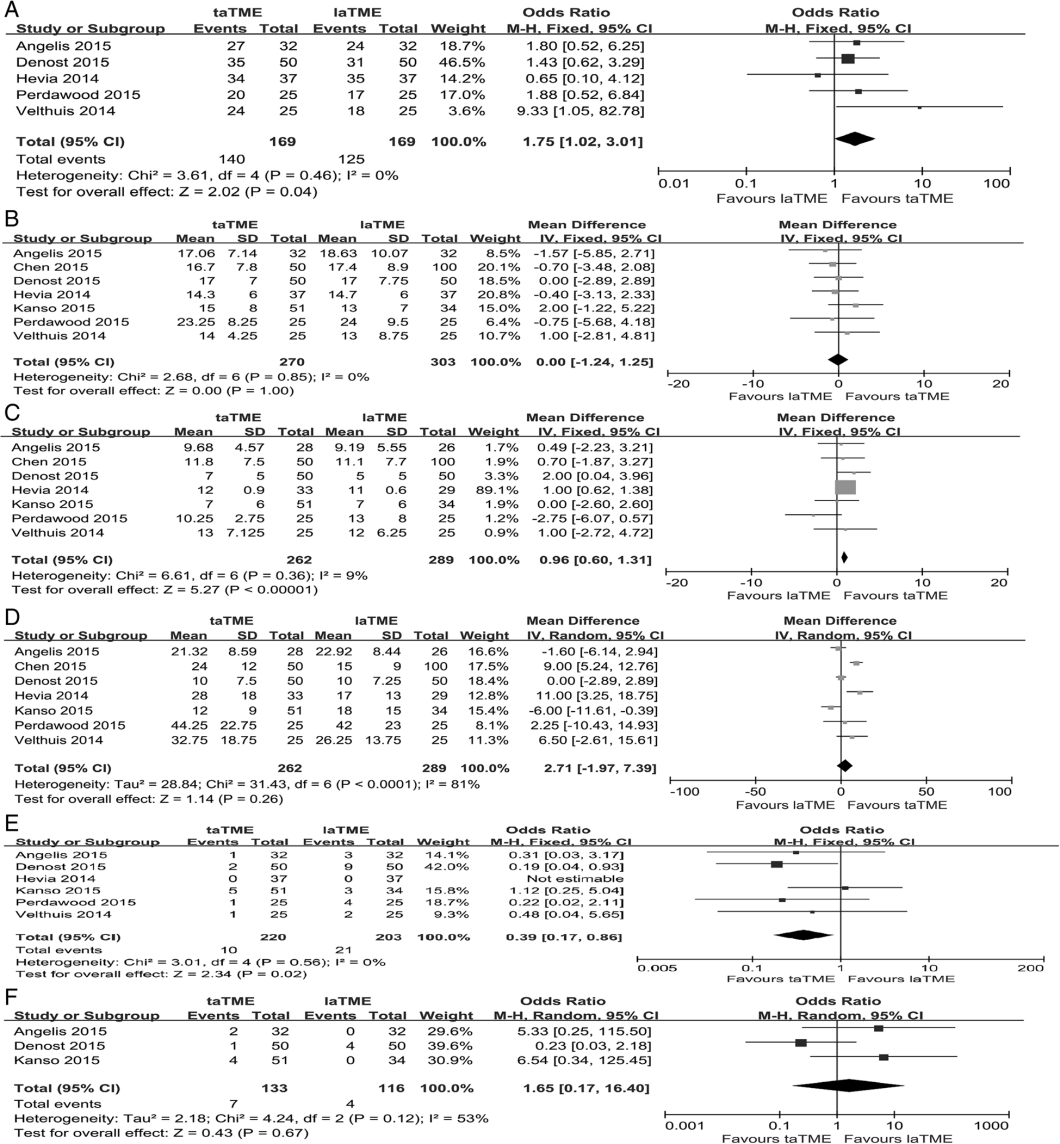
Forest plot based on oncological outcomes (**a**) Mactosocopic quality of mesoretum (**b**) Harvested lymph nodes (**c**) Circumferential resection margin (**d**) Distal resection margin (**e**) Positive circumferential resection margin (**f**) Positive distal resection margin

The quality of the mesorectum was scored using three grades (complete, nearly complete and incomplete), as defined by Quirke [33]. On the basis of this standardized method, five of the studies included reported the macroscopic quality of the mesorectum [26, 28, 29, 31, 32]. After pooled analysis, the complete grade for the quality of the mesorectum was significantly higher for taTME than for laTME (OR = 1.75, 95% CI = 1.02–3.01, *P =* 0.04; Fig. 2a). All the studies included provided information on harvested lymph nodes. The pooled analysis of the seven studies showed that harvested lymph nodes were equivalent between the two groups (WMD = 0.00, 95% CI = –1.24–1.25, *P =* 1.00; Fig. 2b).

With regard to the surgical resection margin, all the studies provided sufficient data on CRM and DRM. Among them, three studies reported patients who achieved complete remission after neoadjuvant treatment [28–30] and two studies evaluated the CRM and DRM only in patients without complete response after neoadjuvant treatment [29, 30]. We excluded the patients with complete remission in these two studies from our overall analysis of the outcomes of CRM and DRM. In the pooled data, the taTME group showed a significantly greater CRM than the laTME group (WMD = 0.96, 95% CI = 0.60–1.31, *P <*0.01; Fig. 2c), but a comparable DRM was observed between the two groups (WMD = 2.71, 95% CI = –1.97–7.39, *P =* 0.26; Fig. 2d). Among the studies, six provided data on positive CRM [26–29, 31, 32] and three on positive DRM [27, 29, 32]. Meta-analysis indicated that a significantly lower number of patients in the taTME group had a positive CRM (OR = 0.39, 95% CI = 0.17–0.86, *P =* 0.02; Fig. 2e), but there was comparable DRM involvement between the two groups (OR = 1.65, 95% CI = 0.17–16.40, *P =* 0.67; Fig. 2f).

Except for the outcomes of DRM and positive DRM, all the other oncological outcomes showed no significant heterogeneity between the groups. Detailed information on the oncological outcomes of included studies is summarized in Table 2.

**Table 2:** Detailed information of oncological and perioperative outcomes of included studies

Studies	Velthuis [31]	Kanso [27]	Hevia [28]	Chen [30]	Denost [32]	Perdawood [26]	Angelis [29]
Mactoscopic quality of mesorectum	*		*		*	*	*
Harvested lymph nodes	*	*	*	*	*	*	*
Circumferential resection margin	*	*	*	*	*	*	*
Positive circumferential resection margin	*	*	*		*	*	*
Distal resection margin	*	*	*	*	*	*	*
Positive distal resection margin		*			*		*
Length of resected specimen	*						*
Complete remission after neoadjuvant			*	*			*
Operative time		*	*	*	*	*	*
Conversion		*	*	*	*	*	*
Hospital stay		*	*	*	*	*	*
Intraoperative complications			*	*		*	*
Postoperative complications		*	*	*	*	*	*
Anastomotic leakage		*	*	*	*	*	*
Ileus			*	*	*	*	*
Acute urinary retention			*	*	*	*	*
Blood loss				*		*	
Mobilization of splenic flexure			*	*		*	
Readmission			*	*		*	*
Mortality		*			*		*
Type of anastomosis		*	*	*		*	
Disease-free survival		*					
Starting diet period			*				
Days to Foley removal				*			
Diverting Ostomy			*	*			

### Perioperative outcomes

**Fig. 3:**
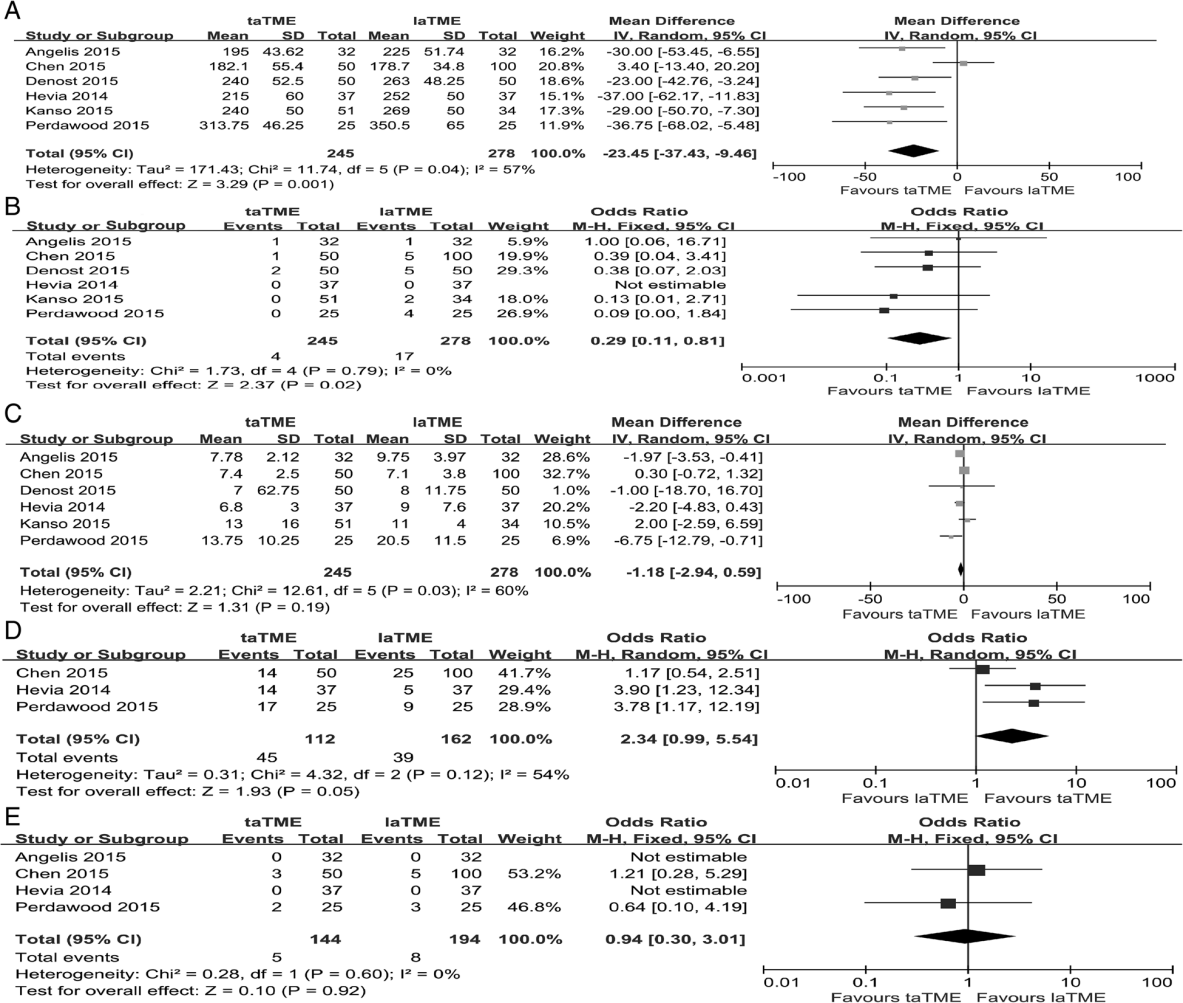
Forest plot based on perioperative outcomes (**a**) Operative time (**b**) Conversion (**c**) Hospital stay (**d**) Mobilization of splenic flexure (**e**) Intraoperative complications

Given that Velthuis et al. [31] only provided results on the pathological characteristics, a meta-analysis was conducted using the remaining six studies to compare the operative and perioperative outcomes between the two groups. In terms of operative outcomes, data on operation time, conversion rate and hospital stay were available for these six studies [26–30, 32]. After pooled analysis, we found that the taTME group showed a significantly shorter operation time (WMD = –23.45, 95% CI = –37.43 to –9.46, *P <*0.01; Fig. 3a), a lower conversion rate (OR = 0.29, 95% CI = 0.11–0.81, *P =* 0.02; Fig. 3b) and a comparable hospital stay (WMD = –1.18, 95% CI = –2.94–0.59, *P =* 0.19; Fig. 3c). Three studies provided data on mobilization of the splenic flexure in the two groups [26, 28, 30] and more mobilization of the splenic flexure was achieved in the taTME group (OR = 2.34, 95% CI = 0.99–5.54, *P =* 0.05; Fig. 3d). In addition, a pooled analysis of intraoperative complications, based on four studies, was conducted [26, 28–30] and there was no difference between the groups for this outcome (OR = 0.94, 95% CI = 0.30–3.01, *P =* 0.92; Fig. 3e). Two studies also indicated that the taTME group showed significantly less blood loss [26, 30] and we did not conduct a pooled analysis because the low number of studies caused considerable heterogeneity.

**Fig. 4:**
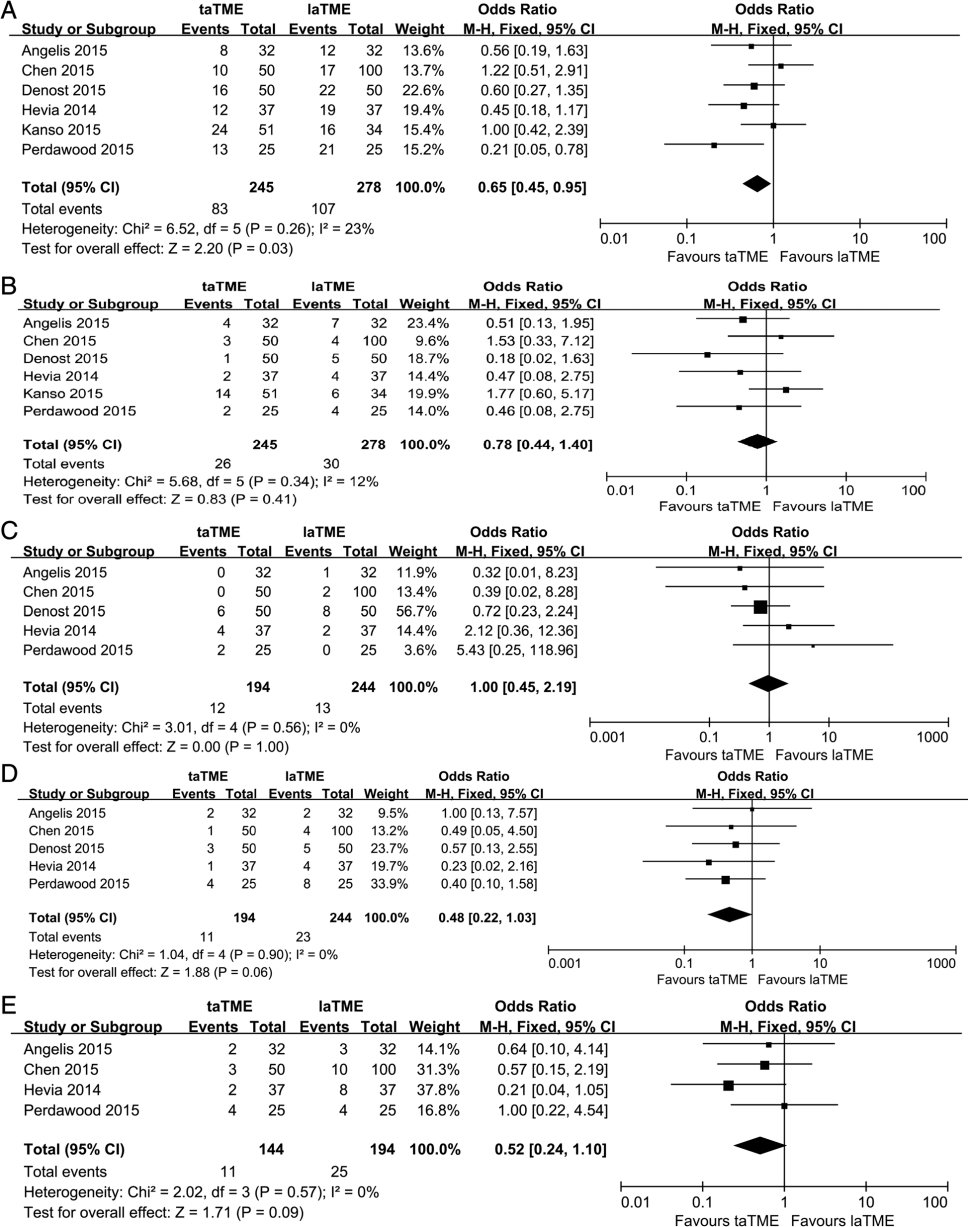
Forest plot based on perioperative outcomes (**a**) Postoperative complications (**b**) Anastomotic leakage (**c**) Ileus (**d**) Urinary morbidity (**e**) Readmission

Regarding the short-term outcomes, all six remaining studies provided information about postoperative complications. In the pooled data, the taTME group showed a significantly lower rate of postoperative complications than the laTME group (OR = 0.65, 95% CI = 0.45–0.95, *P =* 0.03; Fig. 4a). Of note, the occurrence of anastomotic leakage, ileus and urinary morbidity was comparable between the two groups (anastomotic leakage: OR = 0.78, 95% CI = 0.44–1.40, *P =* 0.41; ileus: OR = 1.00, 95% CI = 0.45–2.19, *P =* 1.00; urinary morbidity: OR = 0.48, 95% CI = 0.22–1.03, *P =* 0.06; Fig. 4b–d). In addition, four studies reported the readmission rate [26, 28–30]. A pooled analysis showed a tendency that fewer patients after taTME would require readmission, although this was not statistically significant (OR = 0.52, 95% CI = 0.24–1.10, *P =* 0.09; Fig. 4e).

Except for operative time, hospital stay and molization of splenic flexure, no significant heterogeneity was observed between the groups for other perioperative outcomes. Detailed information on the perioperative outcomes of included stuedies is also summarized in Table 2.

### Subgroup analyses

The term taTME includes two different concepts (partial and total taTME) [34]. Among the studies included in the meta-analysis, two reported the use of conventional retractors to perform a partial taTME [27, 32] the other five studies used a standard transanal access platform to perform a total taTME [26, 28–31]. Hence, to eliminate the heterogeneity introduced by differences in surgical technique, we conducted a subgroup analysis of the oncological and perioperative outcomes, based on total taTME, to further verify our pooled results. Our subgroup analysis showed that the benefits of total taTME were obvious, which was consistent with our overall analysis (Table 3).

**Table 3:** Subgroup analysis based on total taTME

Outcomes	No. of patients		No. of studies	OR/WMD	95%CI	Heterogeneity		*P* value
	taTME	laTME		Low high		I^2^	*P* value	
Oncological outcomes								
Mactoscopic quality of mesorectum	119	119	4	2.03	0.99 4.16	11%	0.34	0.05
Harvested lymph nodes	169	219	5	−0.45	−1.98 1.08	0%	0.93	0.56
Circumferential resection margin	161	205	5	0.94	0.57 1.30	20%	0.29	<0.01^*^
Positive circumferential resectionmargin	119	119	4	0.31	0.08 1.18	0%	0.90	0.09
Distal resection margin	161	205	5	5.48	−0.17 11.13	73%	<0.01	0.06
Positive distal resection margin	83	66	2	5.98	0.71 50.5	0%	0.92	0.10
Perioperative outcomes								
Operative time	144	194	4	−23.29	−45.60 -0.98	72%	0.01	0.04^*^
Conversion	144	194	4	0.31	0.08 1.18	0%	0.90	0.09
Hospital stay	144	194	4	−1.62	−3.62 0.38	74%	0.01	0.11
Intraoperative complications	144	194	4	0.94	0.30 3.01	0%	0.60	0.92
Postoperative complications	144	194	4	0.59	0.35 0.97	45%	0.14	0.04^*^
Anastomotic leakage	144	194	4	0.65	0.30 1.42	0%	0.66	0.28
Ileus	144	194	4	1.37	0.45 4.13	0%	0.49	0.58
Acute urinary retention	144	194	4	0.45	0.18 1.10	0%	0.81	0.08
Mobilization of splenic flexure	112	162	3	2.34	0.99 5.54	54%	0.12	0.05
Readmission	144	194	4	0.52	0.24 1.10	0%	0.57	0.09

*taTME* transanal total mesorectal excision, *laTME* laparoscopic total mesorectal excision, *OR* odds ratios, *WMD* weighted mean difference, *CI* confidence interval; *P vaule with statistical significance

## Discussion

Laparoscopic procedures are generally thought to have better outcomes than open procedures. However, recent two RCTs (AlaCaRT and ACOSOG Z6051) both confirmed that laparoscopic resection failed to meet the criterion for noninferiority for pathologic outcomes when compared with open section for rectal cancer patients [35, 36]. An explanation for this finding is that proctectomy can be very difficult to work in the deep pelvis with in-line rigid instruments from angles that require complicated maneuvers to reach the extremes of the pelvis. Hence, both AlaCaRT and ACOSOG Z6501 indicated that modification of instruments or a different platform such as robotics or taTME will improve efficacy of minimally invasive techniques. Over the last decade, transanal approaches have been extensively used to overcome the inherent shortcomings of laTME [37–39]. Among these emerging transanal techniques, taTME is a new minimally invasive procedure with essential aim of improving oncological treatment quality and avoiding pelvic nerve injury in patients with mid- or low-rectal cancer. Given the encouraging outcomes of systematic investigation of taTME for patients with rectal cancer [40, 41] taTME may be optimized as a surgical approach for rectal cancer. In comparison with conventional laTME, taTME defines the distal resection margin more precisely, with better visualization of the distal rectum, and allows the surgeon to perform the deep pelvic dissection without the need for difficult retraction (even in the deep, narrow male pelvis or in obese patients) [42]. Heald has already stressed the importance of taTME as a new solution to some old problems [43]. However, the benefits of taTME compared with laTME must be confirmed before carrying out multicenter RCTs and unifying taTME procedures. Hence, we conducted this quantitative meta-analysis to investigate whether taTME can show significant benefits with regard to oncological and perioperative outcomes, when compared with laTME.

Based on the results of our meta-analysis for oncological outcomes, we found that patients in the taTME group had a significantly higher rate of complete specimens, longer CRM and less positive CRM involvement. In addition, in terms of perioperative outcomes, the taTME group had significantly shorter operation times and a lower conversion rate. Of note, a significantly lower rate of postoperative complications was observed in the taTME group in comparison with the laTME group. Our findings have provided direct evidence that taTME shows benefits with regard to short-term outcomes for patients with rectal cancer.

Our overall and subgroup analyses both indicated the significant advantages of taTME in achieving complete grading of mesorectal quality. Complete or nearly complete mesorectal fascia is a recognized and universally accepted positive prognostic factor, whereas an incomplete fascia is associated with unfavorable oncological outcomes [44]. Based on the studies included, the percentage of patients with complete mesorectum was 83.4% in the taTME group and 73.4% in the laTME group. In addition, achievement of complete plus nearly complete mesorectum was also greater in the taTME group (95.3% versus 88.2%). Hence, for patients with mid- or low-rectal cancer, taTME may achieve a complete or nearly complete resection of the mesorectum relative easily, compared with laTME. However, whether a higher quality of mesorectal resection will convert into longer survival remains unknown.

The CRM and positive CRM are important indicators of the outcome for patients undergoing TME for rectal cancer [45, 46]. Our results confirmed a significant advantage of taTME in CRM and less positive CRM involvement. However, for the DRM and positive DRM, our results did not reach statistical significance. On one hand, considerable heterogeneity was observed for these two outcomes, which may have been caused by differences in tumor location. In fact, two studies enrolled patients with only low rectal cancer [27, 32], the other five studies enrolled patients with mid- or low-rectal cancer [26, 28–31]. On the other hand, a significant difference in the distance of the tumor from the anal verge was observed in Chen’s study (*P* = 0.022) [30]. Although we could not eliminate the heterogeneity of DRM and positive DRM in our present study, on the basis of the rationale of the dissection in taTME, the potential advantages in these two outcomes justify further study in a large RCT.

With regard to the operative outcomes, taTME and laTME showed comparable results for hospital stay and readmission rate. However, a significantly shorter operation time and lower conversion rate were observed for taTME. One explanation is that taTME can be performed by two teams simultaneously, which obviously decreased the operation time in the pooled analysis [28, 30]. However, it is noteworthy that six of the included studies showed a shorter operation time for the taTME group, irrespective of whether one or two teams were working. The “down-to-up” procedure indeed overcomes the technical limitations of laparoscopy and helps surgeons perform the surgical procedures efficiently. In addition, we assessed the reasons for conversion of the approach. In the taTME group, only one patient underwent conversion to and open approach because of technical difficulty (1/4; 25%), whereas eight patients in the laTME group (8/17; 47%) underwent conversion. The significantly higher conversion rate in the laTME group was primarily due to the difficult pelvic approach in patients with unfavorable characteristics; taTME may overcome these limitations to decrease the incidence of conversion. Furthermore, our results showed a higher rate of moblization of splenic flexure in taTME group. Hence, we want to explore whether use of diverting ostomy may be an affecting factor for moblization of splenic flexure in our present study. Two included studies reported the data of using ostomy between two groups [28, 30]. In study of Hevia et al [28], 86% (32/37) patients in taTME group used diverting ileostomy and 81% (30/37) patients in laTME group (*P* = 0.53). In addition, Chen et al [30] indicated that 92% (46/50) patients in taTME group underwent protective enterostomy in comparison with 91% (91/100) patients in laTME group (*P* = 0.839). Based on this limited data, both groups showed equal rate of using ostomy and we could not get a definite correlation between undergoing ostomy and easier taking down splenic flexure in taTME group. Therefore, the potential factors affecting mobilization of splenic flexure in taTME cases needed to be further explored.

Safety is always the most important issue for a new technique. Our meta-analysis indicated a comparable rate of intraoperative complications and a significantly lower incidence of postoperative complications in the taTME group when compared with the laTME group. The tendency for a lower incidence of postoperative complications in the taTME group may also explain the lower readmission rate for these patients in comparison with the laTME group. However, these results need to be interpreted with caution because they are derived mainly from retrospective studies. Among the types of postoperative complication, our pooled analysis showed that the incidence of anastomotic leakage, ileus and urinary morbidity were comparable between the two groups. In fact, one of the included studies showed a higher incidence of anastomotic leakage in the taTME group [28]. The height of the anastomosis, a risk factor for the development of leakage, may explain this finding [47]. The distance of the tumor from the dentate line varied in the studies included, and was lower in the taTME group (1.6 cm versus 1.8 cm; *P* = 0.11). Of note, an obviously lower incidence of urinary morbidity (infection, dysfunction and retention) was observed in the taTME group, although this did not reach statistical significance. A possible explanation is that taTME provides improved pelvic visualization with enhanced anatomical definition, allowing more accurate dissection through the presacral plane between the mesorectal and pelvic fascia, which may result in sparing of the autonomic nerves during mesorectal dissection, and therefore result in a lower incidence of urinary dysfunction [39, 42]. However, little is known about the long-term quality of life of these patients, or about the risk and the incidence of sexual and urinary dysfunction related to this procedure. Hence, the benefits of taTME with regard to postoperative complications need to be verified by multicenter RCTs.

As the new surgical technique of taTME is adopted increasingly by surgeons, the patient selection criteria will be crucial and will continue to animate debate. Based on the studies included in our meta-analysis, taTME was performed primarily in patients requiring surgical resection for mid- and low-rectal cancer. In addition, taTME may be more suitable for male patients with high BMI and a narrow pelvis. The study of Rouanet et al. [7] also confirmed that taTME is a feasible alternative surgical option to conventional laparoscopy for patients with unfavorable characteristics. Of note, the protocol published recently for a multicenter RCT comparing taTME with laTME (COLOR III) has formulated strict criteria for patient selection [25]. According to the selection criteria of this protocol, T3 tumors with margins <1mm to the endopelvic fascia, tumors with ingrowth in the internal sphincter or m. levator ani and all T4 tumors as staged prior to neoadjuvant therapy were excluded [25]. However, the nature of the surgical candidates best suited to taTME treatment requires further study.

There were some limitations to our present meta-analysis. Except for one study (a RCT by Denost et al.) [32], the other studies included were all retrospective matched case–control trials, which slightly decreases the power of our meta-analysis. In addition, the results on both taTME and laTME were obtained at high volume centers with large minimally invasive proctectomy experiences. Hence, whether the comparable technical success of taTME could be achieved in low volume centers remained further study before this technique fully accepted by surgeon. Furthermore, a standardized procedure and transanal access platform of taTME were not formulated, and differences among the studies in surgical procedure and instruments may have contributed to the heterogeneity in our pooled analysis. Meanwhile, our study only indicated the benefits of taTME in short-term outcomes, compared with laTME; the long-term oncological and functional results should be awaited before completely adopting this new technique. Furthermore, the patients enrolled in our meta-analysis showed inconsistencies with regard to baseline information (age, BMI, neoadjuvant treatments, ASA, pT and pN stage). For example, the patients in the taTME group of the study by Perdawood et al. [26] showed a obviously higher BMI when compared with those in the laTME group (*P* = 0.07), the patients in the study of Hevia et al. [28] showed a difference in age between the two groups (*P* = 0.06), and the distance of tumor the above the anal verge showed a significant difference between groups in Chen’s study (*P* = 0.02) [30]. Importantly, neoadjuvant treatments may be a potential confounding factor for the oncological outcomes of rectal cancer [48]. However, we could not conduct a subgroup analysis based on whether patients had received neoadjuvant treatments before surgical resection by all the possible means. Although the taTME group yielded longer distal margin lengths compared with laTME in the study of Chen et al. [30] (in which all the patients received neoadjuvant chemoradiation before surgery), studies stratified on the basis of neoadjuvant treatment are needed to verify the advantages of taTME.

## Conclusion

Although some limitations existed in present study, our meta-analysis first provides that taTME can achieve comparable technical success in comparison with laTME, in the treatment of rectal cancer. Multicenter RCTs comparing taTME with laTME with long-term outcomes are required to evaluate the efficacy and safety of taTME further as a valid treatment for rectal cancer.

## Abbreviations

ASA, American Society of Anesthesiologists; BMI, body mass index; CI, confidence intervals; CRM, circumferential resection margin; DRM, distal resection margin; laTME, laparoscopic total mesorectal excision; NOS, Newcastle–Ottawa Scale; NOTES, natural orifice transluminal endoscopic surgery; ORs, odds ratios; RCTs, randomized clinical trials; SD, standard deviation; TAMIS, transanal minimally invasive surgery; TATA, transabdominal–transanal; taTME, transanal total mesorectal excision; TME, total mesorectal excision; WMD, weighted mean difference
